# Clinical GBM hybrid artificial intelligence for prescription dose recommendation and outcome prediction after gamma knife radiosurgery treatment: a proof-of-concept

**DOI:** 10.3389/fonc.2026.1837357

**Published:** 2026-05-08

**Authors:** Jheremy S. Reyes, Alexandros Bouras, Ajay Niranjan, L Dade Lunsford, Constantinos G. Hadjipanayis

**Affiliations:** 1 Center for Image-Guided Neurosurgery, Department of Neurological Surgery, University of Pittsburgh Medical Center, Pittsburgh, PA, United States; 2 Computational Neurosurgery Research Group (CIGNS-CRG), Center for Image-Guided Neurosurgery, Department of Neurological Surgery, University of Pittsburgh Medical Center, Pittsburgh, PA, United States

**Keywords:** artificial intelligence, gamma knife radiosurgery, glioblastoma, outcomes, personalized treatments

## Abstract

**Background:**

Outcomes after Gamma Knife radiosurgery (GKRS) for recurrent glioblastoma (GBM) are heterogeneous, and prescription dose selection remains challenging in previously irradiated brain. A data-driven approach that individualizes dose while providing quantitative, patient-specific outcome predictions could improve counseling and follow-up planning.

**Methods:**

We performed a retrospective single-center study of recurrent GBM treated with GKRS at the University of Pittsburgh Medical Center (2014–2024) to develop CGH-AI (Clinical GBM Hybrid Artificial Intelligence). The patient-related clinical variables, biopsy-derived markers, and dosimetric parameters were extracted from the medical records. The primary endpoint was local tumor control, modeled as time to local failure. A lesion-level survival model was developed using a Random Survival Forest and internally validated using patient-level grouped cross-validation. Discrimination and calibration were assessed using Harrell’s concordance index (C-index) and integrated Brier score (IBS). A connected dose recommendation engine evaluated clinically feasible candidate doses per case and selected the dose associated with the most favorable predicted local control profile.

**Results:**

The local control survival model achieved strong patient-level internal validation performance (C-index 0.80; IBS 0.14). Sensitivity analyses incorporating biopsy-derived markers yielded similar performance, supporting robustness of the clinical–tumor–dosimetric feature set. The integrated dose recommendation engine consistently identified prescription doses associated with improved predicted local control while providing interpretable, case-specific local control probabilities and expected local control duration.

**Conclusions:**

CGH-AI integrates survival modeling and dose decision support within the GKRS workflow to recommend individualized prescription dose and to predict local control for recurrent GBM, supporting personalized decision-making and surveillance planning.

## Introduction

**Figure 1:**
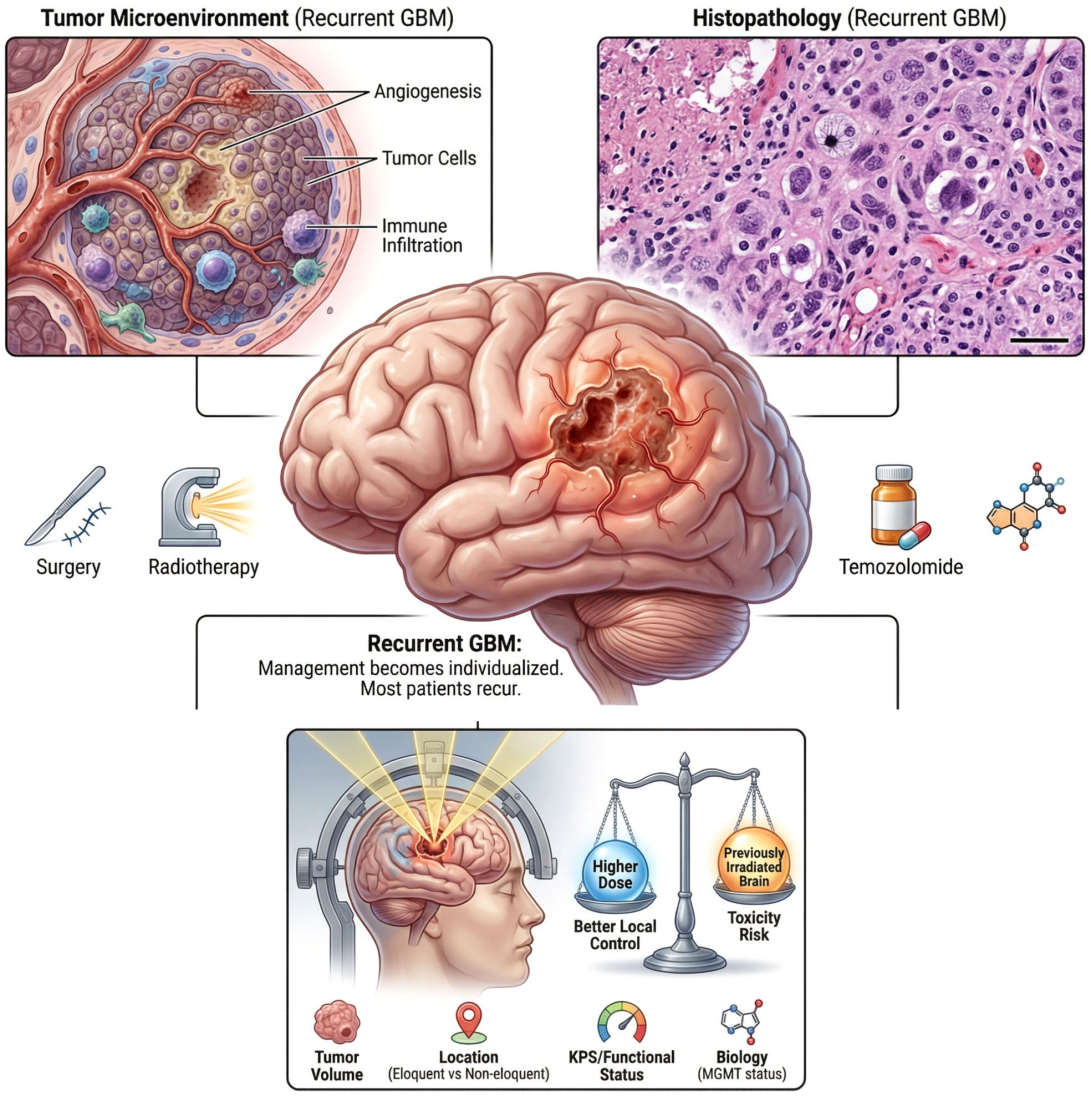
Clinical and biological context of recurrent glioblastoma and the GKRS dose-selection challenge. Recurrent glioblastoma (GBM) is characterized by an aggressive tumor microenvironment with angiogenesis, tumor cell infiltration, and immune involvement, and is defined histopathologically by highly cellular, pleomorphic tumor features. Despite multimodality standard therapy including maximal safe resection, radiotherapy, and temozolomide, most patients experience recurrence and subsequent management becomes individualized. For selected focal recurrences, Gamma Knife radiosurgery (GKRS) is frequently used to deliver highly conformal radiation to the recurrent target while limiting exposure to surrounding brain. However, outcomes after GKRS are heterogeneous, and prescription dose selection requires balancing potential gains in local control with higher dose against toxicity risk in previously irradiated brain. Key factors shaping this trade-off include tumor volume, anatomic location (eloquent vs non-eloquent), functional status (KPS), and tumor biology (e.g., MGMT status).

Recurrent glioblastoma remains one of the most challenging problems in neuro-oncology. Despite maximal safe resection, radiotherapy, and temozolomide-based chemotherapy, most patients experience progression, and subsequent management is highly individualized, shaped by tumor location, prior treatments, neurological function, and the feasibility of additional surgery or systemic therapy (1–6). In this setting, stereotactic radiosurgery with Gamma Knife radiosurgery (GKRS) is frequently considered for selected patients with focal recurrence, offering the potential for durable local control while limiting exposure of surrounding brain to additional radiation (**Figure 1**) (7–13).

However, outcomes after GKRS for recurrent glioblastoma are heterogeneous. Local control and overall survival vary widely across patients, reflecting differences in tumor burden, functional status, imaging-defined target characteristics, dose selection, and the biological aggressiveness of the disease (8–10, 12). Clinically, prescription dose selection is especially difficult because it requires balancing competing priorities: intensifying dose to improve local control versus minimizing the risk of radiation-related toxicity in previously irradiated brain (7–9). Current practice relies heavily on physician experience and institutional patterns, and although general principles exist, there is no widely adopted, data-driven approach that individualizes GKRS dose to patient-specific clinical and tumor features and quantitatively forecasts expected outcomes.

Artificial intelligence (AI) methods—particularly modern survival modeling—provide a framework to address this gap using routinely available treatment-time data (14,-15). Survival models can learn complex, non-linear relationships between baseline variables (clinical, tumor, demographic, and dosimetric factors) and time-to-event outcomes, generating individualized risk estimates and predicted outcome trajectories (16–18). When coupled to a dose-sweep strategy that evaluates predicted outcomes across clinically feasible candidate doses, such models can be extended beyond prognosis toward practical decision support: recommending a prescription dose associated with the best predicted local control profile for a given patient, while simultaneously providing interpretable outcome predictions to inform shared decision-making and follow-up planning (17–21).

Here, we present CGH-AI, a Clinical GBM Hybrid Artificial Intelligence framework designed to (1) predict patient-specific local control after GKRS for recurrent glioblastoma using treatment-time clinical, tumor, demographic, and dosimetric features, and (2) generate a model-based prescription dose decision support that optimizes predicted local control outcomes within a clinically feasible dosing range. We internally validate the survival model using leakage-resistant patient-level splitting to reflect real-world deployment, and we report both discrimination and accuracy to characterize performance. This work aims to provide a reproducible, clinically grounded approach to individualized GKRS decision support in recurrent glioblastoma.

## Methods

### Study design and data source

We performed a retrospective single center cohort study at the University of Pittsburgh Medical Center (UPMC) including patients with recurrent GBM treated with GKRS between 2014 and 2024. Clinical, demographic, tumor, molecular, and radiosurgical planning variables were extracted from institutional records and treatment plans and compiled into a structured dataset. Each record represented a treated radiosurgical target with an associated plan and follow up data. Targets were included if local control status and valid time to event information were available.

### Patient, tumor, and treatment characteristics

Baseline variables included age at treatment, sex, and Karnofsky Performance Status (KPS) at the time of GKRS. Treatment-time, post-plan predictors included clinical variables (age, sex, KPS), tumor variables (contrast-enhancing target volume and anatomic localization), and radiosurgical plan variables (prescription dose and isodose line). Biopsy-derived markers (MGMT and other available molecular fields) were evaluated in sensitivity analyses.

### Treatment protocol

All patients underwent GKRS according to institutional practice. Treatment planning was performed under stereotactic conditions, and prescription dose and isodose selection were determined by the treating teams based on target geometry, proximity to critical structures, prior treatment context, and clinical judgment.

### Outcome and follow up

The primary endpoint was local control. Local failure was defined as radiographic progression of the treated target on serial contrast-enhanced MRI as documented in clinical and radiology reports. Time-to-event was measured from GKRS to the first imaging assessment documenting local failure. Targets without local failure were right-censored at the date of last available imaging follow-up. Cases were excluded if local control status was missing, if the time-to-event or follow-up time was missing or non-positive, or if key treatment-time predictors required for model fitting were unavailable.

### Predictive model development and validation

#### Preprocessing

Continuous variables were converted to numeric format and imputed using median imputation within the training data. Categorical variables were standardized and imputed using the most frequent category, then one-hot encoded. Predictors with no observed values were removed prior to model fitting. All preprocessing was implemented within a single pipeline and learned within each training split to prevent information leakage.

#### Model training

A Random Survival Forest (RSF) was used to model time to local failure under right censoring and to capture non-linear effects and interactions. Hyperparameters were tuned within training folds using an inner cross-validation grid search over the number of trees, maximum depth, minimum samples per split/leaf, and feature subsampling (max_features). IBS was computed over the prespecified evaluation horizon using standard censoring-adjusted estimation based on the training-fold censoring distribution.

#### Internal validation and performance metrics

Internal validation used patient-level grouped cross-validation, such that all observations from a given patient were assigned exclusively to either the training or validation fold in each split. This design was used to minimize leakage and provide deployment-relevant performance estimates. Discrimination was quantified using Harrell’s concordance index (C-index). Overall prediction error was summarized using the integrated Brier score (IBS) over the prespecified evaluation horizon. Confidence intervals for performance metrics were estimated using patient-level resampling (cluster bootstrap and/or repeated grouped cross-validation).

#### Dose recommendation framework

A model-based dose recommendation module evaluated predicted local control across a clinically feasible grid of candidate prescription doses. The recommended dose was defined as the candidate dose associated with the most favorable predicted local control utility (e.g., local control at 12 months or expected local control duration). Recommendations were constrained to the feasible dose range and are presented as decision support within observed practice patterns.

### Statistical analysis and ethics

Descriptive statistics were reported using standard summary measures. All analyses were performed in Python with fixed random seeds for reproducibility. This retrospective study was conducted under institutional oversight per UPMC requirements, with consent waived where applicable.

## Results

### Study cohort

The analytic dataset included 73 patients with recurrent GBM treated with GKRS at UPMC (2014–2024). Median age at diagnosis was 60 years (IQR 55–70; mean 61.8 ± 11.1, range 27–82). Sex distribution was 51 males (69.9%) and 22 females (30.1%). Pre-treatment functional status at GKRS was high, with a median KPS of 80 (IQR 70–90; mean 78.6 ± 11.8, range 40–100) (**Table 1**).

**Table 1:** Baseline clinical, tumor, molecular, and gamma knife radiosurgery (GKRS) treatment characteristics of the study cohort.

Characteristic	Overall (N = 73)
Age at diagnosis, years	60 (55–70); mean 61.8 ± 11.1; range 27–82
Sex	Male 51 (69.9%); Female 22 (30.1%)
KPS before GKRS	80 (70–90); mean 78.6 ± 11.8; range 40–100
MGMT status	Methylated 37 (50.7%); Unmethylated 35 (47.9%)
TERT status	Mutated 33 (45.2%); Wildtype 3 (4.1%)
PTEN status	Mutated 33 (45.2%); Wildtype 14 (19.2%)
Pre-GKRS contrast-enhancing tumor volume, cm³	9.90 (3.39–15.4); mean 10.8 ± 9.71; range 0.12–48.4
Prescription dose, Gy	14 (13–14); mean 13.8 ± 1.51; range 10–18
Prescription isodose line, %	50 (49.6–50); mean 51.8 ± 7.70; range 38.8–80.9
Bevacizumab exposure	Yes 63 (86.3%); No 10 (13.7%)
Enrolled in clinical trial	Yes 7 (9.6%); Not recorded 66 (90.4%)
Local failure events	18/73 (24.7%)
Time from GKRS to local failure	9.58 (5.22–14.8); mean 13.5 ± 16.1; range 3–87.5

Continuous variables are reported as median (interquartile range), with mean ± standard deviation and range. Categorical variables are reported as counts and percentages. Local failure events were defined by documented loss of local control during imaging follow up; targets without local failure were censored at last available imaging assessment. Molecular variables are summarized as recorded in the source dataset, and missingness reflects incomplete availability of pathology and biomarker testing in routine clinical practice.

At the target level, the pre-treatment contrast-enhancing tumor volume had a median of 9.90 cm³ (IQR 3.39–15.36; mean 10.80 ± 9.71, range 0.12–48.35). Prescription dose was median 14.0 Gy (IQR 13.0–14.0; mean 13.76 ± 1.51, range 10.0–18.0). Prescription isodose line was median 50% (IQR 49.6–50.0; mean 51.8 ± 7.7, range 38.8–80.9).

Tumor location at baseline was most commonly frontal (22/72, 30.6%) and temporal (22/72, 30.6%), followed by other/deep midline structures (14/72, 19.4%), parietal (8/72, 11.1%), and occipital (6/72, 8.3%). MGMT status was available for nearly all patients and was methylated in 37/73 (50.7%), and unmethylated in 35/73 (47.9%).

Local failure occurred in 18/73 (24.7%) evaluable cases; 55/73 (75.3%) were censored. Median time from GKRS to local failure or last imaging follow-up was 9.5 months (IQR 5.2–14.1).

### Survival machine learning model performance (local control)

Under patient-level grouped internal validation, the RSF local control model demonstrated good performance (C-index = 0.80, 95% CI 0.77–0.83; IBS = 0.14, 95% CI 0.10–0.18). After preprocessing and one-hot encoding, the final feature matrix comprised 28 predictors (**Table 2**), corresponding to ~0.64 events per encoded predictor (18 events). Given the limited event count relative to the encoded dimensionality, performance should be interpreted as proof-of-concept and subject to uncertainty despite leakage-resistant patient-level validation.

**Table 2:** Encoded predictors used in the final local control survival model (one-hot expanded feature set).

Encoded predictor name	Original variable	Type	Level (if categorical)
num:Age at diagnosis	Age at diagnosis	Numeric	
num:Coverage	Coverage	Numeric	
num:Dose_x_KPS	Dose_x_KPS	Numeric	
num:Dose_x_Vol	Dose_x_Vol	Numeric	
num:Isodose Line (%)	Isodose Line (%)	Numeric	
num:Max (Gy)	Max (Gy)	Numeric	
num:Pre-SRS/RT contrast-enhancing tumor volume in cm3 (incl. necrosis)	Pre-SRS/RT contrast-enhancing tumor volume in cm3 (incl. necrosis)	Numeric	
num:SRS Dose (Gy)	SRS Dose (Gy)	Numeric	
num:KPS before SRS or re-RT for Recurrence	KPS before SRS or re-RT for Recurrence	Numeric	
cat:Anatomic localization (1: frontal, 2: parietal, 3: occipital, 4: temporal, 5: cerebellar, 6: others including deep midline structures)_2	Anatomic localization (1: frontal, 2: parietal, 3: occipital, 4: temporal, 5: cerebellar, 6: others including deep midline structures)	Categorical dummy	2
cat:Anatomic localization (1: frontal, 2: parietal, 3: occipital, 4: temporal, 5: cerebellar, 6: others including deep midline structures)_3	Anatomic localization (1: frontal, 2: parietal, 3: occipital, 4: temporal, 5: cerebellar, 6: others including deep midline structures)	Categorical dummy	3
cat:Anatomic localization (1: frontal, 2: parietal, 3: occipital, 4: temporal, 5: cerebellar, 6: others including deep midline structures)_5	Anatomic localization (1: frontal, 2: parietal, 3: occipital, 4: temporal, 5: cerebellar, 6: others including deep midline structures)	Categorical dummy	5
cat:Anatomic localization (1: frontal, 2: parietal, 3: occipital, 4: temporal, 5: cerebellar, 6: others including deep midline structures)_4	Anatomic localization (1: frontal, 2: parietal, 3: occipital, 4: temporal, 5: cerebellar, 6: others including deep midline structures)	Categorical dummy	4
cat:Anatomic localization (1: frontal, 2: parietal, 3: occipital, 4: temporal, 5: cerebellar, 6: others including deep midline structures)_6	Anatomic localization (1: frontal, 2: parietal, 3: occipital, 4: temporal, 5: cerebellar, 6: others including deep midline structures)	Categorical dummy	6
cat:Chromosome 7+/10- (yes/no)_yes	Chromosome 7+/10- (yes/no)	Categorical dummy	yes
cat:EGFR amplification (ampl/non-ampl)_ampl	EGFR amplification (ampl/non-ampl)	Categorical dummy	ampl
cat:EGFR amplification (ampl/non-ampl)_mut	EGFR amplification (ampl/non-ampl)	Categorical dummy	mut
cat:EGFR amplification (ampl/non-ampl)_unampl	EGFR amplification (ampl/non-ampl)	Categorical dummy	unampl
cat:EGFR amplification (ampl/non-ampl)_unmut	EGFR amplification (ampl/non-ampl)	Categorical dummy	unmut
cat:MGMT (methyl/unmethyl/n.a.)_methyl	MGMT (methyl/unmethyl/n.a.)	Categorical dummy	methyl
cat:MGMT (methyl/unmethyl/n.a.)_unmethyl	MGMT (methyl/unmethyl/n.a.)	Categorical dummy	unmethyl
cat:PTEN (mut/umut)_mut	PTEN (mut/umut)	Categorical dummy	mut
cat:PTEN (mut/umut)_umut	PTEN (mut/umut)	Categorical dummy	umut
cat:Sex (M/F)_M	Sex (M/F)	Categorical dummy	M
cat:TERT (mut/unmut)_mut	TERT (mut/unmut)	Categorical dummy	mut
cat:TERT (mut/unmut)_unmut	TERT (mut/unmut)	Categorical dummy	unmut
cat:Tumor localization (1: cortical (sub-cortical)/2: deep-seated (basal ganglia/thalamus) or midline/3: multifocal)_2	Tumor localization (1: cortical (sub-cortical)/2: deep-seated (basal ganglia/thalamus) or midline/3: multifocal)	Categorical dummy	2
cat:Tumor localization (1: cortical (sub-cortical)/2: deep-seated (basal ganglia/thalamus) or midline/3: multifocal)_3	Tumor localization (1: cortical (sub-cortical)/2: deep-seated (basal ganglia/thalamus) or midline/3: multifocal)	Categorical dummy	3

Continuous predictors (prefixed num:) represent numeric treatment-time variables entered into the model after median imputation within training folds. Categorical predictors (prefixed cat:) represent one-hot encoded indicator variables for specific category levels; for each categorical variable, one level is omitted and serves as the implicit reference category. Engineered interaction terms (dose_x_Vol, dose_x_KPS) were included as additional continuous predictors.

### Sensitivity analysis with biopsy-derived variables

In sensitivity analyses incorporating biopsy-derived molecular markers (including MGMT and other available fields), model performance remained similar to the baseline treatment-time model, indicating no meaningful incremental gain in discrimination or overall error in this cohort.

### Time-based individualized outputs and dose recommendation

**Figure 2:**
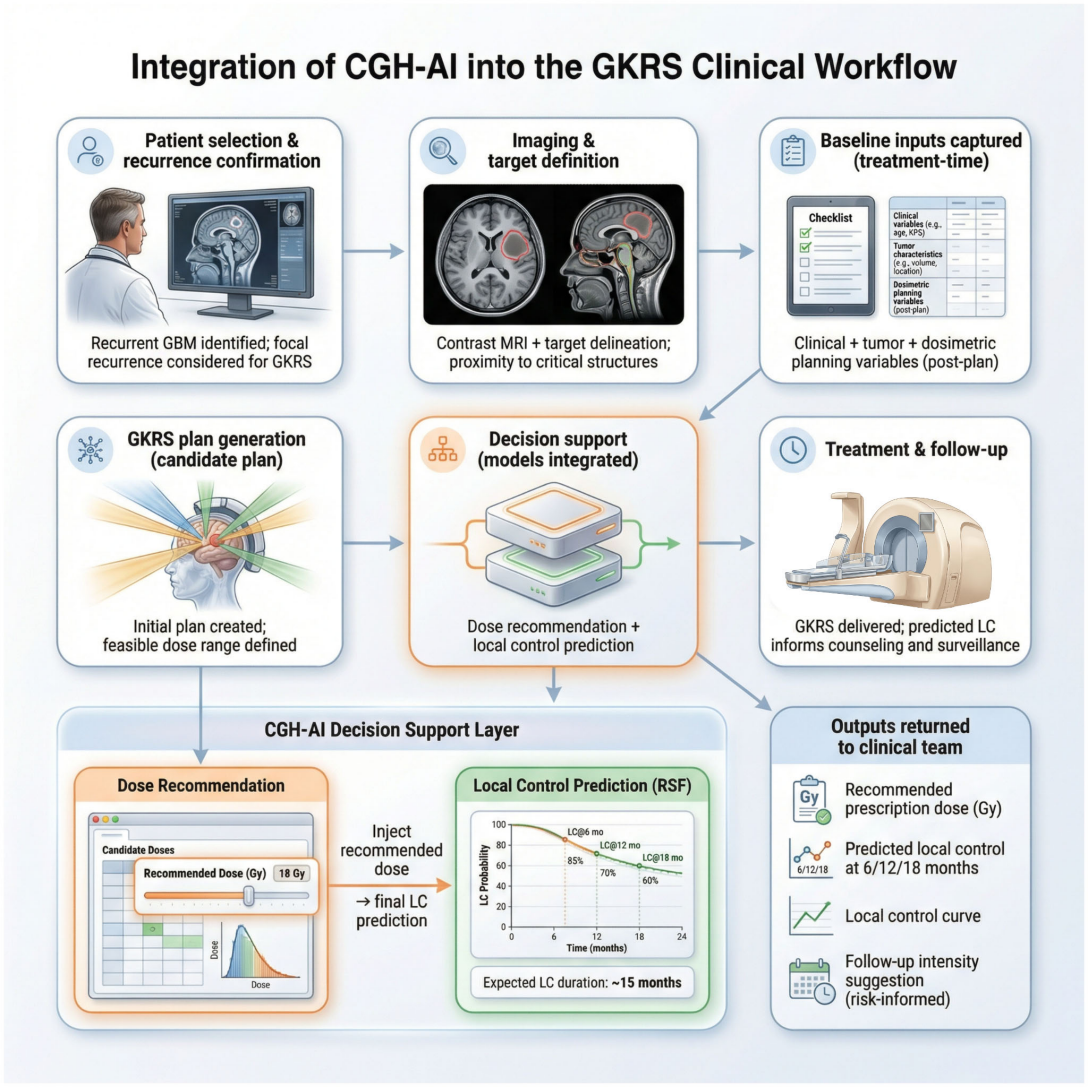
Computational architecture of CGH-AI for dose recommendation and local control prediction after GKRS. The framework begins with a retrospective UPMC recurrent GBM GKRS cohort (2014–2024) and a treatment-time, post-plan feature set spanning clinical (age, sex, KPS), tumor (volume, location, eloquence), and dosimetric variables (prescription dose, isodose line), with optional inclusion of biopsy-derived markers (MGMT, EGFR, PTEN, TERT). A standardized training pipeline performs preprocessing (imputation, one-hot encoding, and removal of all-missing features), followed by patient-level grouped cross-validation (GroupKFold by patient_id) and inner cross-validation hyperparameter tuning to prevent information leakage. Model A is a Random Survival Forest local control survival model that outputs an individualized local control curve and time-based summaries (local control at 6, 12, and 18 months and expected local control). Model B is a dose recommendation engine that evaluates a clinically feasible grid of candidate prescription doses by estimating local control per dose, selects the recommended dose according to a predefined objective, and injects the selected dose into Model A to generate the final case-specific local control predictions. End-to-end outputs include the recommended dose (Gy), predicted local control at prespecified horizons, the local control curve, and exportable ranked dose candidates with a summary per case.

**Figure 3:**
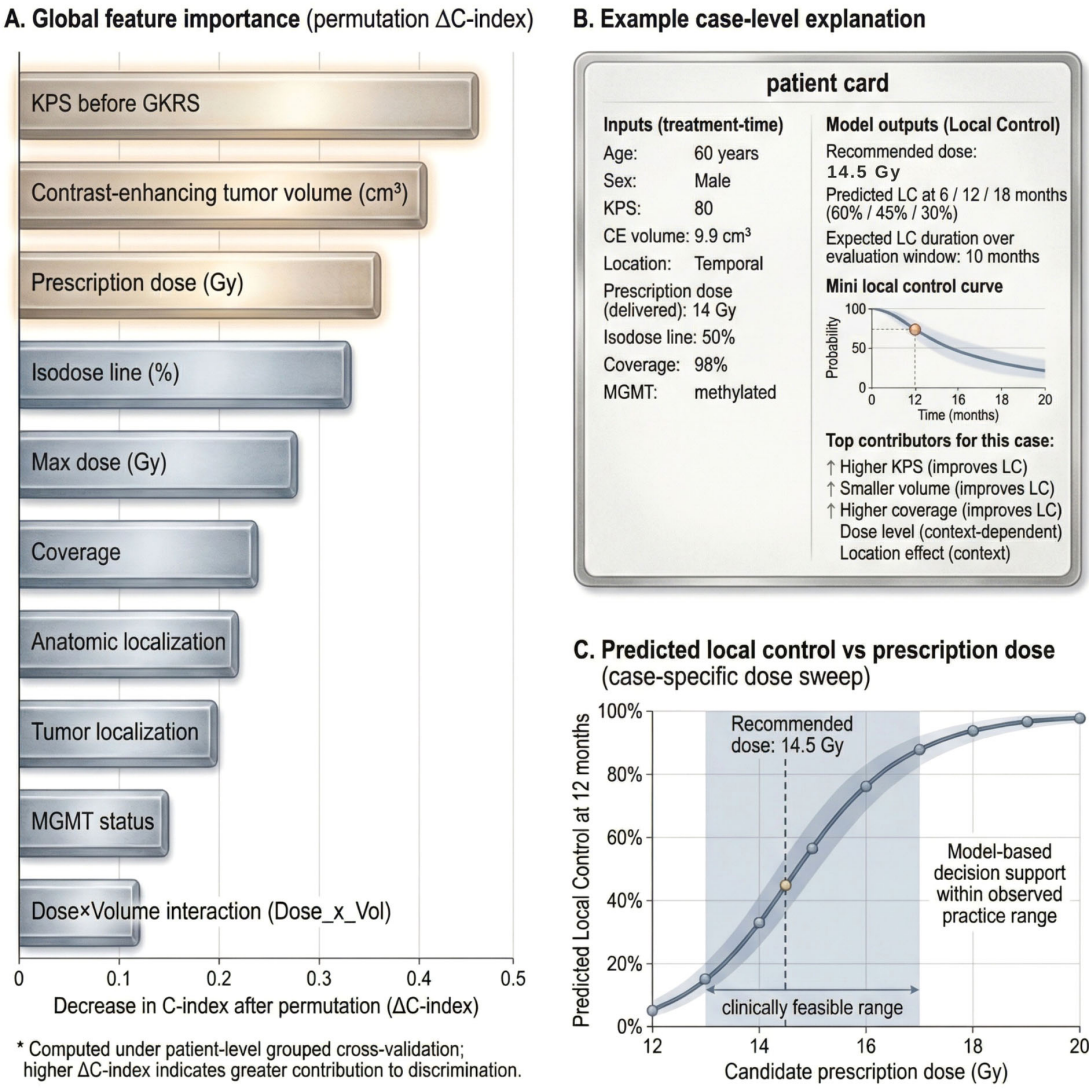
Model explainability for local control prediction after GKRS. **(A)** global permutation importance ranked by the mean decrease in C-index (ΔC-index) when each predictor is permuted under patient-level grouped cross-validation, highlighting the variables most contributing to discrimination. **(B)** example case-level output showing treatment-time inputs and the corresponding local control predictions returned by the model, including local control probabilities at 6, 12, and 18 months and expected local control duration over the evaluation window, along with the top qualitative contributors for that case. **(C)** case-specific dose sweep illustrating predicted local control at 12 months across a clinically feasible prescription dose range, with the recommended dose highlighted to provide transparent, model-based decision support within observed practice constraints.

The survival model generated individualized local control curves and clinically interpretable time-based predictions (e.g., local control probability at standard horizons and expected local control duration over the prespecified horizon). The dose recommendation module successfully integrated with the local control model by evaluating predicted outcomes across a clinically feasible dose grid and selecting the prescription dose associated with the best predicted local control profile, while automatically returning the corresponding individualized local control estimates for the recommended dose (**Figure 2**). Permutation importance identified the strongest contributors to discrimination under patient-level grouped validation (**Figure 3A**). A representative case-level explanation and a case-specific dose sweep illustrate how outputs can be interpreted in clinical use (**Figures 3B, C**).

## Discussion

CGH-AI demonstrates that treatment-time variables routinely available in Gamma Knife workflows can be leveraged to build a clinically usable, end-to-end decision-support pipeline for recurrent glioblastoma. In patient-level grouped internal validation, the local control survival model achieved good performance (C-index 0.80, IBS 0.14), indicating that the framework can meaningfully discriminate targets at higher risk of earlier local failure while providing reasonably accurate time-dependent estimates over the evaluation horizon. This level of performance is particularly relevant in recurrent GBM, where outcomes after GKRS remain heterogeneous and prescription dose selection is often guided by experience and institutional practice rather than a quantitative, individualized framework.

**Figure 4:**
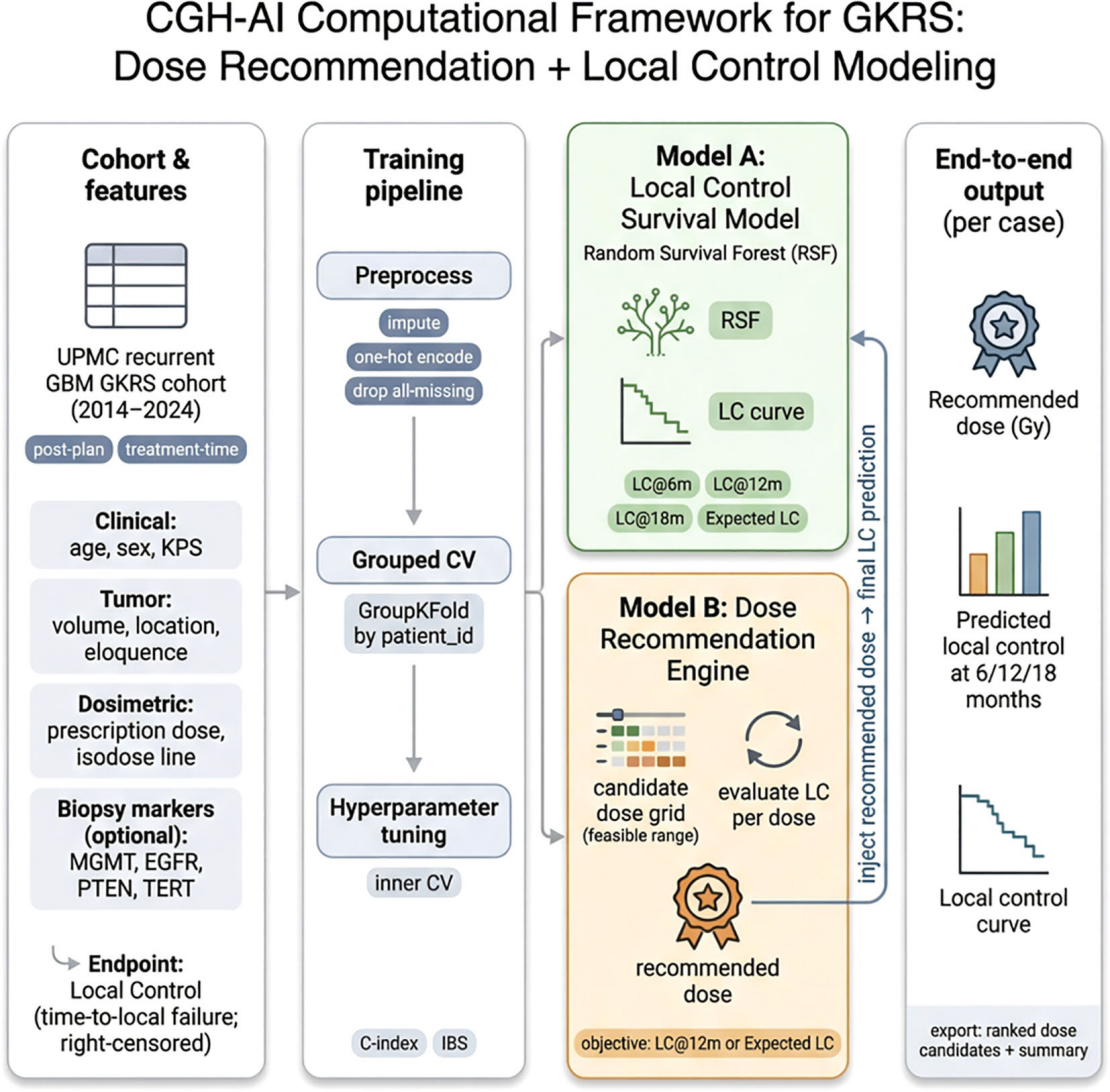
Integration of CGH-AI into the GKRS clinical workflow for recurrent glioblastoma. The workflow begins with patient selection and confirmation of focal recurrence, followed by contrast-enhanced MRI review and target definition. Treatment-time, post-plan variables spanning clinical, tumor, and dosimetric characteristics are captured after initial GKRS plan generation and definition of a feasible prescription dose range. CGH-AI is then applied as a decision-support layer composed of two connected components: a dose recommendation engine that evaluates candidate prescription doses within the feasible range and selects a recommended dose, and a local control survival model (RSF) that generates case-specific local control predictions, including a local control curve, local control probabilities at prespecified horizons (6, 12, and 18 months), and expected local control duration. The recommended dose is injected into the local control model to produce final predictions that are returned to the clinical team.

A central contribution of this study is the connected architecture: dose decision support and outcome prediction are not separate analyses but a single integrated workflow. The system evaluates predicted local control across a clinically feasible grid of candidate prescription doses, selects the dose associated with the most favorable predicted local control profile, and then automatically generates the corresponding individualized local control outputs for the recommended prescription. This “recommend + predict” loop mirrors real planning behavior—considering multiple dose options and their expected trade-offs—and produces outputs that are directly interpretable at the point of care, including local control probabilities at standard horizons and an expected local control duration over the prespecified follow-up window. The exportable artifacts produced by the pipeline (ranked dose candidates and the recommended local control curve) also support auditability and enable prospective evaluation in realistic clinical workflows (**Figure 4**).

The biopsy-derived sensitivity analyses were an important and practical finding. Incorporating molecular and pathology variables commonly reported in GBM, including MGMT and other markers available in the dataset, did not materially change model discrimination or overall error compared with the treatment-time feature set alone. This suggests that, in this cohort, the dominant predictive signal for local control after GKRS was captured by treatment-time clinical, tumor, and dosimetric variables, supporting practical utility in settings where molecular testing may be incomplete or heterogeneously documented at the time of radiosurgical planning.

The lack of measurable performance gain from MGMT and other markers should be interpreted cautiously. Molecular variables were incompletely available and heterogeneously documented, and the limited sample size reduces power to detect incremental effects. Accordingly, these results should not be interpreted as evidence that molecular factors are unimportant for GBM biology or outcomes. Importantly, the framework provides transparent interpretability via global permutation importance and case-level outputs, including a dose-sweep profile, which may facilitate clinical review and adoption.

Taken together, these results support CGH-AI as a clinical proof-of-concept framework for individualized GKRS decision support in recurrent GBM, delivering internally validated local control predictions under leakage-resistant patient-level splitting and prescription dose recommendations explicitly linked to predicted outcomes within observed clinical constraints. By pairing survival modeling with a transparent dose-sweep decision layer, CGH-AI provides a reproducible approach to standardizing dose selection and making outcome expectations more transparent in a setting where clinicians routinely make high-stakes planning decisions under uncertainty. Importantly, dose decisions in recurrent GBM inherently balance local control against toxicity in previously irradiated brain. A key next step is joint modeling of local control and radiation-related toxicity (e.g., radionecrosis/AREs) to enable explicit benefit–risk decision support.

### Limitations

This study has several limitations that should be considered when interpreting the findings. First, the analysis is retrospective and single-center, and treatment decisions (including prescription dose) reflect institutional practice patterns and clinician judgment. As a result, the model may capture center-specific workflows and case selection, and external validation is needed to confirm generalizability across institutions, Gamma Knife platforms, and follow-up practices.

Second, the cohort size and event count are limited (73 patients with 18 local failure events), which constrains model stability and increases the risk of overfitting in flexible survival learners. Although we used leakage-resistant patient-level grouped validation and report uncertainty for performance estimates, confidence intervals may remain wide and performance may be sensitive to the distribution of events and follow-up times. The number of predictors relative to events is an important constraint in this setting, and results should be interpreted as a proof of concept rather than definitive performance.

Third, local control was determined from routine clinical imaging follow-up and documentation. Variability in imaging intervals, interpretation, and documentation can introduce outcome misclassification, and the timing of local failure is interval-based rather than continuously observed. Treatment-related imaging changes in previously irradiated brain may further complicate adjudication. Follow-up duration and censoring patterns can also influence time-dependent metrics, including the integrated Brier score, which is sensitive to the evaluation horizon and the number of patients at risk at later timepoints.

Fourth, several predictors exhibited missingness and heterogeneous coding, particularly biopsy-derived molecular fields and selected dosimetric variables. While the pipeline handled missing values via fold-specific preprocessing, incomplete availability may limit the ability to detect incremental benefit from molecular markers and may reduce robustness in settings with different testing patterns or documentation practices. In addition, several GBM prognostic and treatment-context factors were not uniformly available for modeling, including number of prior recurrences, time from initial diagnosis, extent of resection, and systemic therapy details. These unmeasured confounders may influence both outcome and prescription dose selection.

Fifth, the dose recommendation module is derived from observational data in which prescription dose is not randomly assigned and is influenced by tumor burden, location, prior radiation context, and physician judgment, introducing treatment selection bias and confounding by indication. Accordingly, recommended doses should be interpreted as model-based decision support associated with more favorable predicted outcomes within the observed practice range, rather than causal dose-response claims. Prospective testing is required to assess clinical utility, safety, and concordance with physician planning.

Finally, this study focused on local control and did not explicitly model competing endpoints such as radionecrosis or other radiation-related toxicity, overall survival, or broader intracranial and systemic progression patterns that also influence management decisions. Future work incorporating multi-endpoint modeling, toxicity-aware optimization, and prospective workflow evaluation would strengthen clinical translation. External validation, including temporal validation on later cases and multi-center validation across practice patterns, is required prior to clinical deployment.

## Conclusions

CGH-AI is an end-to-end decision-support framework for recurrent GBM treated with GKRS that links model-based prescription dose decision support to individualized local control prediction. The local control survival model showed good leakage-resistant patient-level internal validation (C-index 0.80; IBS 0.14). These results support CGH-AI as a practical, reproducible approach for personalized GKRS planning and motivate external validation and prospective evaluation.

## Data Availability

The raw data supporting the conclusions of this article will be made available by the authors, without undue reservation.
